# Prevention of the Quality Degradation of Antarctic Krill (*Euphausia superba*) Meal through Two-Stage Drying

**DOI:** 10.3390/foods13111706

**Published:** 2024-05-29

**Authors:** Yao Zheng, Shuaishuai Zhang, Liu Yang, Banghong Wei, Quanyou Guo

**Affiliations:** 1 Key Laboratory of Oceanic and Polar Fisheries, Ministry of Agriculture and Rural Affairs, East China Sea Fisheries Research Institute, Chinese Academy of Fishery Sciences, Shanghai 200090, China; zhengyao@ecsf.ac.cn (Y.Z.); z15239471039@126.com (S.Z.); yangliuedu@126.com (L.Y.); weibanghong@ecsf.ac.cn (B.W.); 2 Laoshan Laboratory, Qingdao 266200, China; 3 School of Health Science and Engineering, University of Shanghai for Science and Technology, Shanghai 200093, China

**Keywords:** krill meal, two-stage drying, hot-air drying, vacuum drying, bioactive compounds

## Abstract

To achieve a krill meal of high quality, a two-stage drying involving hot-air drying and vacuum drying was investigated. Five experimental groups were established according to the different drying conditions in the second stage, including 95 °C and 101 kPa, 95 °C and 60 kPa, 75 °C and 101 kPa, 75 °C and 60 kPa, and 75 °C and 20 kPa. The results showed that reducing the drying temperature and vacuum pressure in the second stage had a significant impact on the drying characteristics, sensory quality, and bioactive compounds of krill meal. Among all five groups, the drying condition of 75 °C and 60 kPa maintained a high drying rate while preserving a phospholipid content of 30.01 mg/kg and an astaxanthin content of 37.41 mg/kg. It also effectively reduced the isomerization of astaxanthin and the oxidation of unsaturated fatty acids. These results suggested that the two-stage drying method may contribute to the production of high-quality krill meal.

## 1. Introduction

Antarctic krill (*Euphausia superba*) is regarded as one of the most abundant animal species on the planet, making up an estimated biomass of around 379 million tonnes [1]. Considering the characteristics of long-distance pelagic fishing and the high activity of endogenous enzymes, Antarctic krill is required to be processed onboard as soon as possible after catching to avoid blackening, autolysis, and other quality deterioration [2]. Currently, krill meal is the most dominant product processed onboard as a result of the reduction in transportation costs and an improvement in storage stability by removing a large amount of water. Owing to the high abundance of protein and other bioactive compounds, such as astaxanthin, ω-3 polyunsaturated fatty acids (PUFAs), and α-tocopherol, krill meal can be directly utilized as animal feed [3] or further processed into high-value products for food, cosmetics, and health products purposes [4,5].

The processing of krill meal mainly involves cooking, solid–liquid separation, drying, pulverization, and sieving [4]. Among these, drying is considered the most essential stage, during which heat transfer and water diffusion are accomplished. Hot-air drying is one of the most commonly used methods in the production of krill meal, during which dehydration is achieved by convection. However, the heat-labile bioactive compounds are prone to degrade or lose their biological activity during this process [6]. It was reported that about 7%, 18%, and 60% decrease in astaxanthin was observed during drying at 40, 60, and 100 °C, respectively [7]. In contrast, vacuum drying, a drying method conducted below the standard atmospheric pressure, has been widely applied in materials containing heat-sensitive substances [8]. More vitamin C content in orange slices was observed by vacuum drying compared to hot-air drying [9]. Chlorogenic acid, the main active ingredient of *Lonicera japonica* Thunb, was also effectively retained by using vacuum drying [10]. Nevertheless, vacuum drying always consumes more energy compared to hot-air drying due to the requirement of creating a negative pressure environment.

In order to reduce energy consumption and enhance product quality simultaneously, a combined two-stage drying is now often adopted. The quality changes in food have been investigated through two-stage drying by combining different drying conditions or methods [11]. Better quality in terms of sensory, nutrition, and cell structure was achieved in bamboo shoot slices through two-stage drying involving hot-air and vacuum freeze-drying while also reducing energy consumption by about 21% [12]. Similar results were also found in strawberries [13], ginger [14], and button mushrooms [15]. Due to the specific conditions of shipboard processing of krill meal, certain advanced drying methods like freeze-drying have not been implemented. The predominant drying methods currently employed in this context are hot-air drying and vacuum drying. However, studies on the two-stage drying of Antarctic krill meal are relatively limited, particularly regarding its effects on typical bioactive compounds such as astaxanthin.

Based on our previous research, the optimal range for drying temperatures and vacuum pressure, as well as the water transition point, have been determined. The aim of this study was to apply a combination of hot-air drying and vacuum drying to krill meal and evaluate the effects of different drying conditions in the second stage on drying characteristics, sensory quality, and bioactive compounds. The findings of this research will contribute to the production of high-quality krill meal, providing valuable materials for the development of high-value-added products.

## 2. Materials and Methods

### 2.1. Materials

Frozen Antarctic krill samples were obtained from China National Fisheries Corporation (Beijing, China). The fresh krill was harvested in the FAO 48 area in the Antarctic Ocean by ‘Longfa’ fishing vessels in June 2022 and stored at −30 °C for transportation. Upon arrival at our laboratory in March 2023, the krill samples were kept at −80 °C until use.

The standard substances of astaxanthin with a purity of 99% were purchased from Dr. Ehrenstorfer Co., Ltd. (Augsburg, Germany). Trichloroacetic acid (TCA), trichloromethane, ethylene diamine tetraacetic acid (EDTA), thiobarbituric acid were purchased from Shanghai Macklin Biochemical Technology Co., Ltd. (Shanghai, China). Other reagents were of analytical grade.

### 2.2. Sample Preparation

**Figure 1:**
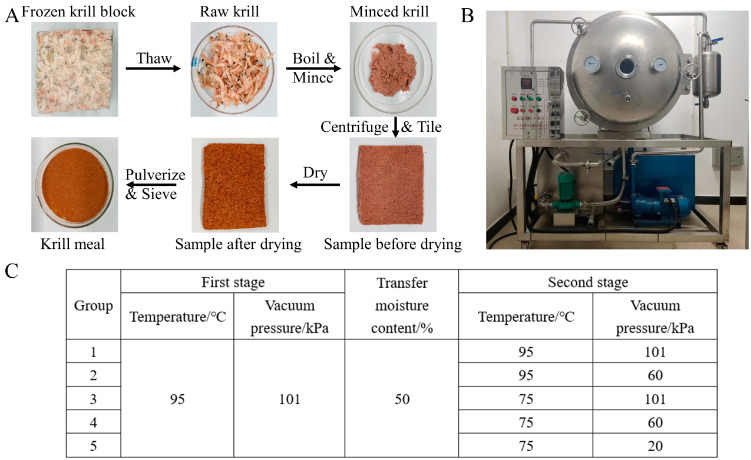
A flow diagram of krill meal preparation (**A**); the physical picture of the drying equipment (**B**); experimental design (**C**).

The preparation of the Antarctic krill meal was conducted by simulating the manufacturing process onboard (Figure 1A). Briefly, eight hundred grams of frozen krill block was thawed with running water for 2.5 h at room temperature, and the krill samples were boiled for 10 min to inactivate the endogenous enzyme. After mincing for 1 min, about 460 g of samples were uniformly tiled on the flat plate with a thickness of 5 mm for the drying process (YZG-600, Plate Dryer, Weifang, China, Figure 1B). A two-stage convective drying method was adopted in this study, and the drying conditions in the first stage were kept at 95 °C under the standard atmospheric pressure (101 kPa). Five experimental groups were conducted according to the different drying conditions in the second stage: (1) Group 1, 95 °C and 101 kPa; (2) Group 2, 95 °C and 60 kPa; (3) Group 3, 75 °C and 101 kPa; (4) Group 4, 75 °C and 60 kPa; (5) Group 5, 75 °C and 20 kPa (Figure 1C). The second stage was initiated when the moisture content reached 50 ± 1%, and the drying procedure was terminated when the moisture content decreased to 10 ± 1%. The converting moisture point and other parameters were selected based on the results of our previous experiments, which indicated that bioactive compounds began to degrade rapidly when the moisture content reached about 50%.

### 2.3. Drying Characteristics

Drying characteristics are reflected by moisture ratio (MR) and drying rate (DR) based on the method described in previous research [6]. The dimensionless MR of the krill meal was calculated by the following equation:

$$\mathrm{MR}=\frac{M_{t}}{M_{e}}$$

where *M_t_* is the moisture content at *t* time of drying, g/g, and *M*_0_ is the initial moisture content, g/g. All moisture content was expressed on a dry basis.

The drying rate of the krill meal was calculated by the following equation:

$$\mathrm{DR}=\frac{M_{t1}-M_{t2}}{t_{2}-t_{1}}$$

where *M_t_*_1_ and *M_t_*_2_ represented the moisture content of krill meal at times *t*_1_ and *t*_2_, respectively, and *t*_1_ and *t*_2_ represented the corresponding drying time, h. The dimension of DR is g·g^−1^·h^−1^.

### 2.4. Low-Field Nuclear Magnetic Resonance (LF-NMR)

Water distribution was measured by an NMR Analyzer (PQ001-20-25V, Niumag Corporation, Suzhou, China), following a previously described method with minor modifications [16]. Five grams of krill meal samples were placed in NMR tubes, and the transverse relaxation time (T2) was determined with the Carr–Purcell–Meiboom–Gill (CPMG) pulse sequence. The relevant parameters were set as follows: SF = 22 MHz; NECH = 5000; TE = 0.2 ms; TW = 2000.00 ms; and NS = 4. The decay curves were measured for continuous multi-exponential using MultiExp Inv analysis software (Version 4.0, Niumag Corporation, Suzhou, China).

### 2.5. Color Analysis

The krill meal samples were evenly spread in a powder testing box, and their color was measured using a portable colorimeter (CR-400, Konica Minolta, Tokyo, Japan). The color was represented using the CIE-Lab scale as L* (lightness), a* (greenness or redness), and b* (yellowness or blueness).

### 2.6. Headspace Gas Chromatography–Ion Mobility Spectrometry (HS-GC-IMS)

Volatile compounds in the krill sample were analyzed using HS-GC-IMS (FlavourSpec^®^, Gesellschaft für Analytische Sensorsysteme mbH, Dortmund, Germany) following a previously established method with some modifications [17]. A quantity of 0.11 g of the krill sample was weighed and incubated at 40 °C for 20 min with a rotation speed of 500 rpm. The injection volume was 500 μL, and the injection needle temperature was set to 65 °C. The analysis employed a strong polar chromatographic column (MXT-WAX, 30 m–0.53 mm) operating at 60 °C, and nitrogen gas was used as the carrier gas at a flow rate of 150 mL/min. The analysis time was set to 30 min, and the IMS detector was maintained at a temperature of 45 °C.

### 2.7. Thiobarbituric Acid-Reactive Substances (TBARS)

TBARS (mg MDA/kg) was measured according to the method of Zheng et al. [18]. TBARS were determined at 532 nm (Alpha-1500, Shanghai Puyuan Instrument Co., Ltd., Shanghai, China) and calculated from a standard curve of 1,1,3,3-tetraethoxypropane.

### 2.8. Phospholipid

Phospholipid content was determined through the molybdenum blue spectrophotometric method [19]. Briefly, 1.00 g samples were mixed with 10 mL methanol and 5 mL chloroform. After extracting with ultrasound (JP-100S, Skymen, Shenzhen, China) treatment for 30 min and centrifuging (Avanti J-301, Beckman Coulter, Brea, CA, USA) at 4000 r/min for 5 min, the supernatant was evaporated at 60–80 °C and ashed at 550 °C for 2 h using 0.5 g of zinc oxide. After dissolving in 10 mL of HCl solution (1:1), the solution was heated to a gentle boil for 5 min and then filtered. The solution was neutralized by adding HCl and KOH dropwise, and the volume was subsequently adjusted to 100 mL using water. After mixing with 4 mL of hydrazine sulfate solution (0.015%) and 1 mL of sodium molybdate solution (2.5%), the absorbance was measured at 650 nm. The phospholipid content was calculated by the following equation:

$$phospholipid\;content\;(mg/g\;DW)=\frac{C}{m}\times f\times 26.31$$

where *C* is the content of phosphorus (mg); *m* is the dry basis mass of the sample (g); *f* is the dilution multiple; and 26.31 is the conversion factor used to convert phosphorus content to phospholipid content.

### 2.9. Astaxanthin and the Geometrical Isomers

The extraction and determination of astaxanthin was performed according to the previous method with some modifications [7]. In this method, saponification was employed to convert astaxanthin esters, including monoesters and diesters, into their free form, enabling the measurement of the total astaxanthin content in the sample. Briefly, four grams of anhydrous magnesium sulfate and 10 mL of acetone were added to 10 g of minced krill (krill meal samples were mixed with acetone in a 1:10 ratio, *w*/*v*). After the ultrasound treatment at 15 °C for 15 min, the mixtures were centrifuged at 8000 r/min for 5 min. The above extraction steps were repeated one more time, and the supernatants were combined. Two milliliters of the extract was fully mixed with the 2.9 mL of 20 mmol/L NaOH-methanol and sealed with nitrogen. After incubation for 12 h, 0.1 mL of 0.6 mol/L phosphate-methanol was added and fully mixed. The final solution was filtered using a 0.22 μm membrane filter before HPLC analysis.

The content of astaxanthin and its isomers was determined through an HPLC system (1260, Agilent, Santa Clara, CA, USA) equipped with a C30 column (250 × 4.6 mm, 5 μm) and a UV detector. The mobile phase consisted of methanol (A), methyl tert-butyl ether (B), and phosphoric acid solution (C). The linear gradient was as follows: 15–30% buffer B for 15 min; 30–80% buffer B for 8 min; 80% buffer B for 4 min; 80–15% buffer B for 3 min; and 15% buffer B for 5 min (buffer C remained at 4% throughout). The HPLC conditions were as follows: a flow rate of 1.0 mL/min; column temperature of 25 °C; injection loop of 20 µL; detection λ = 474 nm.

### 2.10. Fatty Acid

The analysis of fatty acid was performed according to the previous method with some modifications [20]. After the extraction of fatty acid and the preparation of fatty acid methyl esters, the fatty acid profile was analyzed by a gas chromatograph (GC) (Agilent 6890N, Agilent Technologies, Santa Clara, CA, USA) fitted with an Agilent DB-23 (0.25 mm × 30 m × 0.25 μm) (Agilent Technologies Inc., USA) and a flame ionization detector (FID). The GC conditions were as follows: a flow rate of 0.6 mL/min; injection loop of 1 µL; column temperature of 45 °C for 2 min; 45 to 105 °C at 25 °C/min for 2 min; 105 to 200 °C at 15 °C/min for 10 min; 200 to 210 °C at 1 °C/min for 10 min; and 201 to 220 °C at 2 °C/min for 10 min.

### 2.11. Statistical Analysis

Three replicated trials were performed for each experiment, and the data were expressed as the mean values ± standard deviations (SPSS 22.0, SPSS Inc., Chicago, IL, USA). Significant differences were identified at the 5% level using Tukey’s test.

## 3. Results and Discussion

### 3.1. Drying Characteristics

#### 3.1.1. Moisture Ratio and Drying Rate

**Figure 2:**
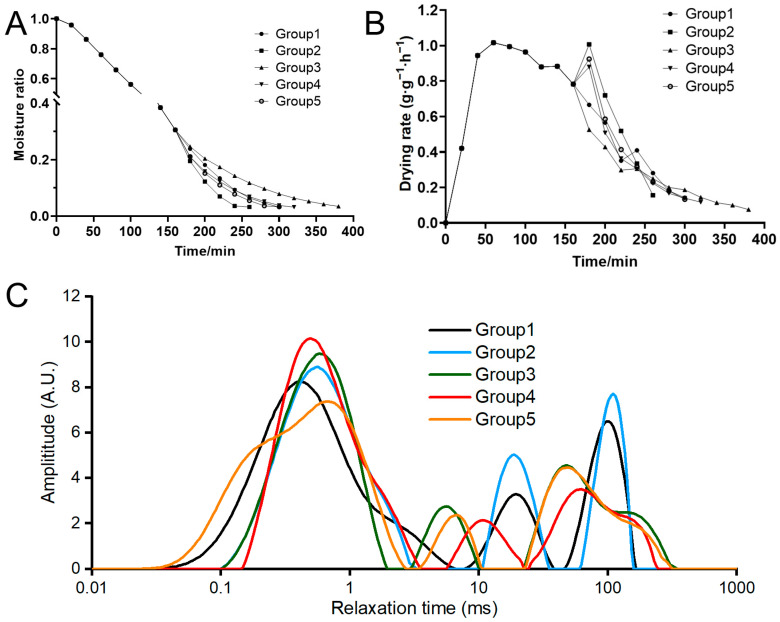
Changes in the moisture ratio (**A**), drying rate (**B**), and water distribution (**C**) of krill meal during two-stage drying. Group 1: dried at 95 °C and 101 kPa; Group 2: dried at 95 °C and 60 kPa; Group 3: dried at 75 °C and 101 kPa; Group 4: dried at 75 °C and 60 kPa; and Group 5: dried at 75 °C and 20 kPa during the second stage.

Drying kinetics studies provide information that helps to understand the drying behavior under different conditions, including temperature and vacuum pressure [21]. The moisture content of krill meal before drying was 76.77% ± 0.23, and at the end of drying, it was 9.86% ± 0.09, 9.79% ± 0.41, 9.69% ± 0.41, 9.74% ± 0.15, and 9.95% ± 0.30 in Groups 1 to 5, taking 300 min, 260 min, 380 min, 320 min, and 300 min, respectively. The moisture ratio and drying rate were calculated to reflect the drying characteristics of krill meal at different two-stage drying conditions (Figure 2A,B). During the entire drying process, the moisture ratio of the krill meal decreased with the increase in drying time. As indicated by Groups 1 and 3, higher temperatures led to accelerated drying rates. This can be attributed to the increased vapor pressure caused by higher temperatures, enabling more efficient removal of internal moisture within a shorter timeframe [21]. Compared to Group 1, which kept the same drying conditions in the second stage, the moisture ratio decreased more rapidly in Group 2, indicating that the decrease in vacuum pressure accelerated the drying process. A higher water rehydration rate was also found in apple slices using vacuum drying [22]. For vacuum drying, the vapor saturation temperature decreases as the vacuum pressure decreases. Consequently, moisture can evaporate at lower temperatures, which leads to an acceleration of mass transfer [6]. The longest drying process of 380 min was observed in Group 3 as the drying temperature decreased from 95 °C to 75 °C, while in Group 4 and Group 5, the prolonged drying process could be counteracted by reducing the vacuum pressure. For the drying rate of all five groups, three distinct periods can be clearly identified, starting with a short rapid period, followed by a constant period, and finally ending with a long falling drying rate period, which is consistent with the results of most studies [23]. In addition, a fluctuation was found in 180 min due to the change in drying conditions. In brief, the drying rate followed the order of Group 2 > Group 1 = Group 5 > Group 4 > Group 3.

#### 3.1.2. Water Distribution

To further evaluate the migration and distribution of water molecules, LF-NMR analysis was carried out. The relaxation time reflects the degree of interaction between water molecules and protein structures. As shown in Figure 2C, all five groups exhibited three distinct peaks, which corresponded to bound water (0.1–10 ms), immobile water (10–100 ms), and free water (100–1000 ms), respectively. Bound water refers to water tightly bound to macromolecules; immobile water refers to water trapped in the cytoplasm, and free water refers to water presented in the vacuoles and intercellular spaces [24]. Based on the peak areas, the immobile water had the highest proportion before drying (specific data not provided), while the bound water had the highest proportion at the end of the drying process. This indicated that the free water and immobile water were abundantly removed during the drying process. Similar results were obtained by monitoring the changes in water distribution during the drying process of mulberries [25]. In Group 1, the relaxation times of immobile and free water were found to be higher compared to the other four groups, suggesting a higher level of water mobility. This could be attributed to the higher temperature maintained consistently throughout the drying process and the absence of vacuum conditions, which significantly disrupted the structure of the muscle cell membrane and myofibrillar protein [26]. Some researchers also argued that drying technology could change the proportion of free hydrogen ions in the material, and a novel drying method could act only on the surface of the material, as proved by scanning electron microscopy [26].

### 3.2. Sensory Quality

#### 3.2.1. Color

**Figure 3:**
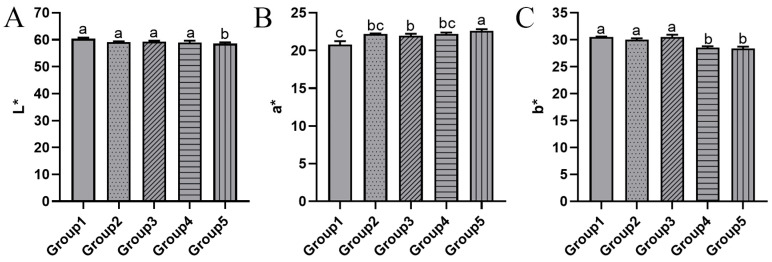
The L* (**A**), a* (**B**), and b* (**C**) values of krill meal under different two-stage drying conditions. Group 1: dried at 95 °C and 101 kPa; Group 2: dried at 95 °C and 60 kPa; Group 3: dried at 75 °C and 101 kPa; Group 4: dried at 75 °C and 60 kPa; and Group 5: dried at 75 °C and 20 kPa during the second stage. The different lowercase letter indicates that the means are significantly different at *p <* 0.05.

Color serves as a crucial sensory parameter for evaluating the quality of krill meal, as it can visually reflect quality differences. As depicted in Figure 3A, there were no significant differences in L* (lightness) values among all groups except for Group 5. In Group 3, where the drying temperature was reduced by 20 degrees compared to Group 1, the a* (redness/greenness) value significantly increased from 20.77 to 21.96 (Figure 3B). As the vacuum pressure was further reduced to 20 kPa in Group 5, the a* value continued to increase significantly, reaching 22.59. The trend of the b* (yellowness or blueness) value is roughly opposite to that of the a* value, with a significant decrease observed in Groups 4 and 5. During storage, krill meal can undergo a color transformation from pale pink or brick red to orange or brown. Therefore, a higher a* value always indicates a better color appearance. Similar results were reported in shrimp (*Penaeidae*) when comparing two drying conditions: drying at a constant temperature of 140 °C throughout the process versus lowering the temperature to 50 °C in the second stage [27]. The latter condition led to a significant increase in the a* value, indicating a good quality of dried shrimp. The color change in krill is influenced by multiple factors, such as the Maillard reaction, degradation of astaxanthin, and lipid oxidation [28]. In summary, the two-stage drying process can effectively preserve the color of krill meal.

#### 3.2.2. Volatile Odor

To examine the odor profile of krill meal under different two-stage drying conditions, the volatile organic compound (VOC) fingerprint was analyzed using GC-IMS. As shown in Table 1, a total of 37 volatile organic compounds were detected, including 9 aldehydes, 5 ketones, 5 alcohols, 11 esters, 3 olefins, 1 alkane, 2 benzene derivatives, and 1 furan derivative. Aldehydes, being the main products of lipid oxidation, significantly contribute to the odor of dried products and have distinct odor characteristics with low odor thresholds [17]. Compared to Group 1, the other four groups showed lower levels of both (E)-2-hexenal and hexanal, indicating that the two-stage drying process could inhibit lipid oxidation to some extent. Ketone compounds are formed through the thermal oxidation of polyunsaturated fatty acids. The content of 2-heptanone, which is an oxidation product of linoleic acid [29], decreased with lower vacuum pressure. Similar results were also observed in Singer (*Tricholoma matsutake*), confirming that high-temperature conditions can result in the formation of aldehydes and ketones during the drying process [30]. Additionally, several short-chain alcohols and hydrocarbon compounds were detected during the analysis. However, their contribution to the overall flavor of krill meal was minimal due to their relatively high odor thresholds [31].

### 3.3. Bioactive Compounds

#### 3.3.1. Lipid Oxidation and Phospholipid Content

**Figure 4:**
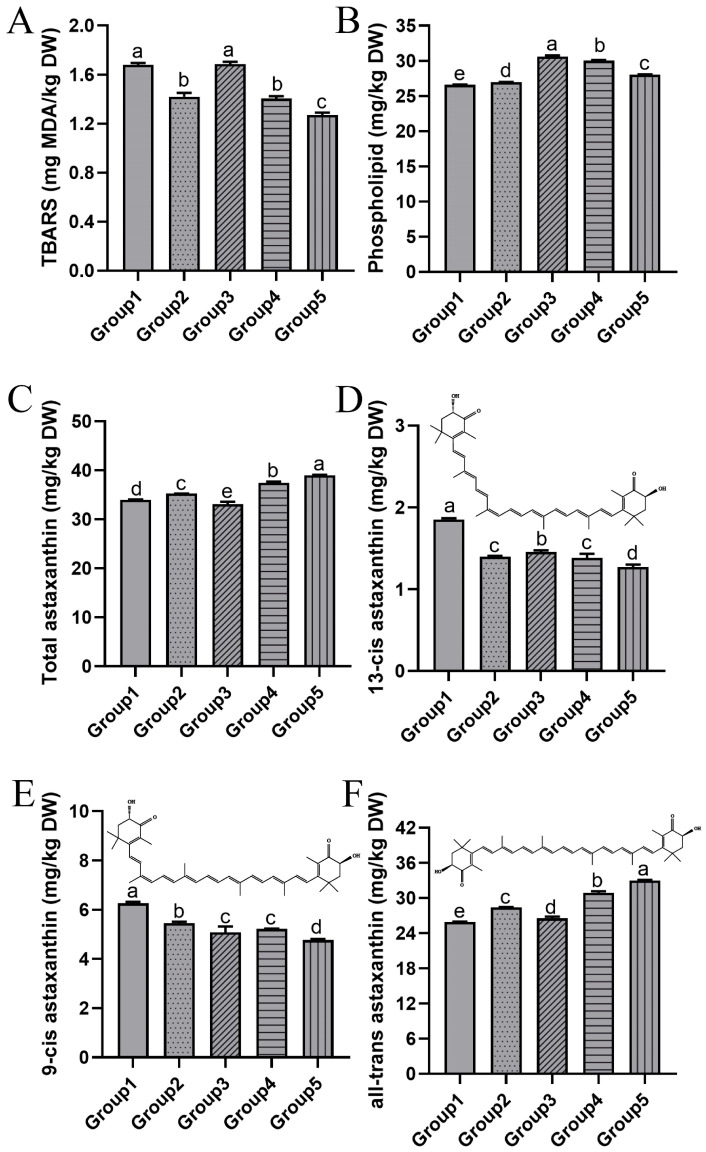
The TBARS (**A**), phospholipid content (**B**), total astaxanthin content (**C**), 13-cis astaxanthin (**D**), 9-cis astaxanthin (**E**), and all-trans astaxanthin (**F**) of krill meal under different two-stage drying conditions. Group 1: dried at 95 °C and 101 kPa; Group 2: dried at 95 °C and 60 kPa; Group 3: dried at 75 °C and 101 kPa; Group 4: dried at 75 °C and 60 kPa; and Group 5: dried at 75 °C and 20 kPa during the second stage. The different lowercase letter indicates that the means are significantly different at *p <* 0.05.

As the secondary product of lipid oxidation, TBARS is commonly used as an indicator to evaluate the degree of lipid oxidation. The lipid oxidation of krill meal under different two-stage drying conditions is illustrated in Figure 4A. Prior to drying, the initial TBARS of krill meal was measured to be 0.92 ± 0.01 mg MDA/kg. A significant increase in TBARS was observed in all five groups, indicating the occurrence of lipid oxidation during the drying process. The presence of heat and oxygen during drying rendered the lipid molecules susceptible to oxidation, particularly in aquatic products rich in unsaturated fatty acids [18]. Among the five groups, Groups 2, 4, and 5 exhibited significantly lower TBARS compared to Groups 1 and 3, suggesting that lipid oxidation was partially inhibited by lower vacuum pressure. A lower TBARS value was also found in chicken slices dried under the vacuum condition [32]. This could be attributed to a decrease in the interaction between oxygen molecules and lipid molecules as the vacuum pressure decreased. Additionally, the increased drying rate induced by vacuum pressure may have reduced the exposure period to high temperatures (as discussed in Section 3.1.1). Consequently, the degree of lipid oxidation was correspondingly reduced.

The phospholipid content of krill meal under different two-stage drying conditions is shown in Figure 4B. The phospholipid content was measured to be 56.60 ± 0.33 mg/kg before drying and decreased to 46.96%, 47.61%, 54.34%, 52.86%, and 49.49% in Groups 1 to 5, respectively. Krill is characterized by its high phospholipid content compared to other aquatic products. In krill oil, it has been documented that nearly half of the eicosapentaenoic acid (EPA) and docosahexaenoic acid (DHA) are located in phospholipids instead of triglycerides [33]. Phospholipid EPA and DHA exhibit better bioavailability, but they are also more susceptible to oxidation. Contrary to the results observed for TBARS, Groups 3, 4, and 5 retained a higher amount of phospholipids, indicating that temperature had a greater impact on phospholipid degradation compared to the vacuum pressure. It was also reported that the high temperature during hot-air drying significantly decreased the contents of phosphatidylcholine and phosphatidylethanolamine in shrimp (*Penaeus vannamei*) [34].

#### 3.3.2. Astaxanthin and Geometrical Isomers

The high content of the highly antioxidant astaxanthin is another notable characteristic of krill meal. As depicted in Figure 4C, the astaxanthin content of Group 1 was 33.99 mg/kg, whereas Group 4 and 5 showed a significant increase of 9.83% and 14.77%, respectively. This indicated that lower temperature and vacuum pressure effectively mitigated the degradation of astaxanthin. Previous studies have also reported a decrease of approximately 17% in astaxanthin content in krill exposed to thermal treatment [35]. Likewise, storage conditions involving low temperatures (−20 °C, 4 °C) and vacuum packaging have been shown to enhance the stability of astaxanthin [36]. It should be noted that astaxanthin molecules with a 3-hydroxy, 4-keto group at the terminal end of their structure are prone to instability during food processing [37]. Krill meal currently serves two primary purposes: (1) as a coloring agent in the aquaculture of salmon; and (2) as an antioxidant for the production of cosmetics and other products [5]. Both of these applications rely on the content of astaxanthin, making it crucial to minimize the degradation of astaxanthin during the drying process.

Furthermore, the astaxanthin molecule contains conjugated double bonds with electrons pi (π) offshore, leading to the presence of geometric isomers such as 13-cis astaxanthin, 9-cis astaxanthin, and all-trans astaxanthin. As shown in Figure 4D, Group 1 exhibited the highest content of 13-cis astaxanthin at 1.84 mg/kg, followed by Groups 2 to 4, with the lowest content observed in Group 5 at 1.30 mg/kg. The content of 9-cis astaxanthin was approximately similar to that of 13-cis astaxanthin (Figure 4E). In terms of all-trans astaxanthin, Group 1 displayed the lowest content, accounting for only 78.53% of the highest group with the drying condition of 75 °C and 20 kPa (Figure 4F). Most astaxanthin/carotenoid naturally exists in a trans structure, which is thermodynamically more stable than the cis structure [5]. Therefore, lower temperatures and vacuum pressure can better preserve the natural structure of astaxanthin in krill meal. A similar conversion from trans to cis form during the drying process was reported by Cong et al. [7]. Isomerization occurs because the polyene chain is susceptible to thermal degradation and oxidation [37]. Interestingly, it has been documented that the cis form, particularly 9-cis astaxanthin, exhibited higher antioxidant activity than the trans form, as demonstrated in in vitro experiments [38].

#### 3.3.3. Fatty Acid Composition

The fatty acid composition of krill meal under different two-stage drying conditions is illustrated in Table 2. A total of 31 fatty acids were identified, including 12 saturated fatty acids, 8 monounsaturated fatty acids (MUFA), and 11 polyunsaturated fatty acids (PUFA). The two-stage drying process demonstrated the ability to retain a higher proportion of unsaturated fatty acids, particularly DHA. A similar phenomenon was observed in krill meal during the manufacturing and drying process [7,35]. It has been widely acknowledged that PUFA can help reduce the risk of various diseases, such as arteriosclerosis, coronary heart disease, inflammatory diseases, and possibly behavioral disorders [39]. As mentioned above, krill meal was rich in phospholipid PUFA. However, the composition and structure of krill lipids make them highly susceptible to oxidation during meal processing, leading to a loss of nutritional value and organoleptic characteristics.

## 4. Conclusions

In this study, a two-stage drying process was employed for Antarctic krill. The drying temperature and vacuum pressure were adjusted when the moisture content reached 50%. Compared to maintaining a constant temperature of 95 °C and vacuum pressure of 101 kPa throughout the entire drying process (Group 1), reducing the drying temperature and vacuum pressure in the second stage can significantly alter the drying characteristics, sensory quality, and bioactive compounds of krill meal. Group 2 achieved a 13.33% increase in drying rate by reducing the vacuum pressure. Group 4 was able to achieve a drying rate comparable to that of Group 1 by simultaneously reducing the drying temperature and vacuum pressure. The two-stage drying process could effectively preserve the a* value of krill meal and reduce the formation of volatile odor compounds like (E)-2-hexenal. It also resulted in better preservation of bioactive components such as phospholipids, astaxanthin, and unsaturated fatty acids, particularly in Group 4 and Group 5. Considering both the production efficiency and the product quality, a drying temperature of 75 °C and a vacuum pressure of 60 kPa can be considered favorable parameters for the second stage of drying. Finally, the current methods for assessing krill meal quality are time-consuming and inefficient. There is an urgent need to develop a rapid detection method. Building upon this, real-time monitoring of drying parameters and krill meal quality can be achieved, enabling dynamic control of the drying process. Moreover, the degradation of active substances during krill meal drying can be mitigated by antioxidants. However, further research is required to investigate the types and methods of antioxidant addition.

## Figures and Tables

**Table 1:** The peak areas of volatile organic compounds of krill meal under different two-stage drying conditions.

	Group 1 ^1^	Group 2	Group 3	Group 4	Group 5
Isoterpinene	855.90 ± 37.80 ^a^	738.92 ± 5.69 ^c^	714.52 ± 4.77 ^c^	801.92 ± 13.64 ^b^	834.57 ± 16.48 ^ab^
(Z)-4-Heptenal	1009.52 ± 192.24 ^b^	1443.57 ± 61.42 ^a^	624.74 ± 56.90 ^c^	1479.70 ± 55.92 ^a^	1414.23 ± 118.57 ^a^
3-Methyl-1-butanol	483.13 ± 78.14 ^b^	585.61 ± 49.56 ^a^	304.79 ± 14.96 ^c^	578.99 ± 19.64 ^a^	505.20 ± 20.41 ^ab^
Dipentene	333.93 ± 14.08 ^a^	239.21 ± 2.92 ^c^	174.44 ± 8.86 ^d^	295.05 ± 4.25 ^b^	231.35 ± 8.19 ^c^
Ethyl pyruvate	4355.27 ± 254.52 ^a^	1920.54 ± 297.64 ^b^	837.80 ± 33.54 ^c^	1794.61 ± 125.21 ^b^	902.98 ± 95.78 ^c^
2-Heptanone	87.04 ± 3.40 ^a^	49.79 ± 3.14 ^c^	26.14 ± 2.78 ^e^	64.89 ± 2.97 ^b^	42.02 ± 4.80 ^d^
Cyclopentanone	905.99 ± 172.49 ^c^	712.11 ± 42.15 ^cd^	625.54 ± 93.29 ^d^	1425.16 ± 206.51 ^a^	1163.76 ± 36.88 ^b^
(E)-2-Hexenal	1110.55 ± 114.23 ^a^	492.55 ± 56.71 ^b^	260.48 ± 5.65 ^c^	449.55 ± 22.67 ^b^	287.10 ± 25.99 ^c^
1,4-Dimethylbenzene	190.58 ± 18.00 ^a^	152.83 ± 10.81 ^b^	99.10 ± 2.39 ^d^	151.58 ± 13.06 ^b^	126.25 ± 9.39 ^c^
1-Butanol	1080.13 ± 27.95 ^a^	629.69 ± 43.73 ^c^	432.41 ± 19.72 ^e^	696.39 ± 12.33 ^b^	483.42 ± 7.88 ^d^
3-Pentanol-D	271.40 ± 29.71 ^c^	176.73 ± 14.33 ^d^	190.60 ± 28.09 ^d^	558.30 ± 77.09 ^a^	373.16 ± 35.60 ^b^
3-pentanol-M	1234.49 ± 58.02 ^c^	992.25 ± 2.98 ^d^	1059.99 ± 81.58 ^d^	1630.35 ± 86.05 ^a^	1477.30 ± 52.50 ^b^
Hexanal	462.53 ± 2.71 ^b^	467.78 ± 27.39 ^b^	367.05 ± 20.92 ^c^	514.24 ± 28.66 ^a^	433.66 ± 17.68 ^b^
Valeraldehyde	4415.02 ± 206.50 ^b^	3734.59 ± 138.59 ^c^	3665.15 ± 56.64 ^c^	4762.56 ± 79.13 ^a^	4551.34 ± 40.84 ^ab^
Ethyl 3-methylbutanoate	3023.46 ± 41.55 ^a^	2610.81 ± 33.00 ^c^	2313.58 ± 22.93 ^d^	2772.85 ± 45.67 ^b^	2568.92 ± 30.23 ^c^
2-Butanol	4672.94 ± 164.85 ^d^	9381.98 ± 81.96 ^a^	8302.70 ± 64.61 ^b^	3807.24 ± 64.77 ^e^	4868.30 ± 72.52 ^c^
Propyl acetate	1491.92 ± 40.23 ^a^	1428.60 ± 7.25 ^b^	1471.18 ± 7.89 ^ab^	1458.11 ± 23.00 ^ab^	1478.96 ± 26.23 ^a^
2,3-Butanedione	675.95 ± 11.33 ^d^	765.77 ± 26.62 ^c^	972.39 ± 9.19 ^a^	750.76 ± 21.88 ^c^	923.63 ± 9.78 ^b^
3-Methylbutyraldehyde	1135.22 ± 30.08 ^d^	1451.21 ± 78.33 ^c^	1466.66 ± 67.44 ^c^	1721.58 ± 32.08 ^b^	1875.72 ± 19.12 ^a^
Ethyl butyrate	771.71 ± 12.38 ^a^	573.87 ± 42.49 ^b^	415.06 ± 19.00 ^d^	588.59 ± 11.85 ^b^	472.96 ± 20.70 ^c^
Butyraldehyde-D	239.14 ± 12.05 ^c^	403.45 ± 52.14 ^b^	418.12 ± 35.51 ^b^	615.52 ± 34.72 ^a^	666.28 ± 20.67 ^a^
Butyraldehyde-M	3402.20 ± 51.56 ^d^	3564.81 ± 43.11 ^c^	5169.60 ± 125.41 ^a^	2844.71 ± 46.93 ^e^	3970.59 ± 113.30 ^b^
1-Octene	38,295.01 ± 647.40 ^a^	20,848.17 ± 2509.28 ^b^	10,536.48 ± 293.09 ^c^	22,992.20 ± 1185.91 ^b^	12,301.65 ± 670.54 ^c^
Butylcyclohexane	2133.51 ± 41.50 ^d^	3672.78 ± 111.23 ^a^	3739.73 ± 18.39 ^a^	2399.09 ± 54.49 ^c^	2844.88 ± 66.76 ^b^
2-Pentanone	350.30 ± 24.46 ^c^	359.85 ± 32.83 ^c^	460.76 ± 7.83 ^b^	449.34 ± 21.30 ^b^	566.55 ± 10.35 ^a^
Ethyl 2-methylbutyrate	235.26 ± 11.46 ^b^	249.22 ± 3.68 ^b^	357.30 ± 1.04 ^a^	250.88 ± 7.86 ^b^	360.95 ± 15.86 ^a^
Tetrahydrofuran	543.02 ± 16.58 ^d^	864.71 ± 90.58 ^c^	1652.08 ± 15.32 ^a^	852.56 ± 59.94 ^c^	1515.79 ± 23.49 ^b^
2-Methylpropanal	490.48 ± 23.08 ^c^	975.51 ± 7.42 ^a^	506.19 ± 7.24 ^c^	720.19 ± 121.01 ^b^	549.83 ± 7.03 ^c^
Methyl acetate	196.88 ± 7.36 ^a^	50.29 ± 4.42 ^e^	61.45 ± 6.36 ^d^	133.91 ± 5.66 ^c^	164.28 ± 3.78 ^b^
Ethyl formate-D	59.21 ± 7.14 ^b^	58.12 ± 21.87 ^b^	34.64 ± 1.52 ^c^	185.00 ± 8.15 ^a^	180.06 ± 0.44 ^a^
Ethyl formate-M	739.14 ± 48.19 ^b^	623.60 ± 115.93 ^c^	433.65 ± 26.49 ^d^	1108.99 ± 15.72 ^a^	1097.49 ± 30.99 ^a^
Ethyl 2-methylpropanoate	179.98 ± 5.56 ^b^	156.14 ± 8.47 ^c^	151.80 ± 3.67 ^c^	178.80 ± 11.52 ^b^	208.12 ± 8.83 ^a^
4-Methyl-3-penten-2-one	126.50 ± 13.34 ^d^	130.76 ± 0.63 ^cd^	149.63 ± 3.06 ^ab^	141.47 ± 7.70 ^bc^	161.68 ± 4.85 ^a^
(E)-2-Pentenal	243.51 ± 7.90 ^b^	222.87 ± 6.69 ^c^	258.84 ± 3.44 ^ab^	245.67 ± 16.64 ^b^	275.55 ± 15.13 ^a^
Ethyl 2-hydroxypropanoate	349.17 ± 8.98 ^a^	304.17 ± 11.89 ^c^	297.52 ± 3.02 ^c^	326.76 ± 3.31 ^b^	334.94 ± 11.10 ^ab^
Butyl acetate	257.81 ± 35.74 ^a^	163.34 ± 0.79 ^b^	92.82 ± 4.46 ^c^	174.91 ± 2.64 ^b^	110.68 ± 7.02 ^c^
4-Isopropyltoluene	41.58 ± 4.91 ^a^	40.92 ± 1.53 ^a^	38.48 ± 0.91 ^a^	40.80 ± 3.67 ^a^	44.19 ± 2.33 ^a^

^1^ Group 1: dried at 95 °C and 101 kPa; Group 2: dried at 95 °C and 60 kPa; Group 3: dried at 75 °C and 101 kPa; Group 4: dried at 75 °C and 60 kPa; and Group 5: dried at 75 °C and 20 kPa during the second stage. The different lowercase letter in the same row indicates that the means are significantly different at *p* < 0.05.

**Table 2:** The fatty acid composition of krill meal under different two-stage drying conditions (g/100 g).

	Group 1 ^1^	Group 2	Group 3	Group 4	Group 5
c12:0	0.02 ± 0.00 ^a^	0.02 ± 0.00 ^a^	0.02 ± 0.00 ^a^	0.01 ± 0.00 ^c^	0.02 ± 0.00 ^b^
c13:0	0.01 ± 0.00 ^b^	0.01 ± 0.00 ^b^	0.01 ± 0.00 ^a^	0.01 ± 0.00 ^c^	0.01 ± 0.00 ^b^
c14:0	1.18 ± 0.00 ^b^	1.15 ± 0.00 ^b^	1.25 ± 0.03 ^a^	0.92 ± 0.01 ^d^	1.09 ± 0.00 ^c^
c15:0	0.04 ± 0.00 ^a^	0.04 ± 0.00 ^b^	0.04 ± 0.00 ^a^	0.03 ± 0.00 ^d^	0.04 ± 0.00 ^c^
c16:0	2.17 ± 0.01 ^b^	2.12 ± 0.01 ^c^	2.28 ± 0.02 ^a^	1.72 ± 0.01 ^e^	1.98 ± 0.01 ^d^
c17:0	0.18 ± 0.00 ^b^	0.18 ± 0.00 ^c^	0.22 ± 0.00 ^a^	0.14 ± 0.00 ^d^	0.16 ± 0.00 ^a^
c18:0	0.14 ± 0.00 ^b^	0.13 ± 0.00 ^c^	0.14 ± 0.00 ^a^	0.11 ± 0.00 ^e^	0.13 ± 0.00 ^d^
c20:0	0.01 ± 0.00 ^b^	0.01 ± 0.00 ^b^	0.00 ± 0.00 ^a^	0.00 ± 0.00 ^c^	0.00 ± 0.00 ^b^
c21:0	0.00 ± 0.00 ^ab^	0.00 ± 0.00 ^bc^	0.01 ± 0.00 ^c^	0.00 ± 0.00 ^d^	0.00 ± 0.00 ^a^
c22:0	0.20 ± 0.02 ^a^	0.25 ± 0.02 ^a^	0.22 ± 0.07 ^a^	0.25 ± 0.02 ^a^	0.28 ± 0.02 ^a^
c23:0	0.00 ± 0.00 ^a^	0.00 ± 0.00 ^a^	0.00 ± 0.00 ^a^	0.00 ± 0.00 ^a^	0.00 ± 0.00 ^a^
c24:0	0.02 ± 0.00 ^b^	0.02 ± 0.00 ^c^	0.02 ± 0.00 ^a^	0.02 ± 0.00 ^e^	0.02 ± 0.00 ^d^
c14:1n5	0.04 ± 0.00 ^b^	0.04 ± 0.00 c	0.04 ± 0.00 ^a^	0.03 ± 0.00 ^e^	0.04 ± 0.00 ^d^
c15:1n5	0.01 ± 0.00 ^b^	0.01 ± 0.00 ^c^	0.01 ± 0.00 ^a^	0.01 ± 0.00 ^e^	0.01 ± 0.00 ^d^
c16:1n7	0.71 ± 0.00 ^b^	0.69 ± 0.00 ^b^	0.77 ± 0.00 ^a^	0.55 ± 0.00 ^c^	0.65 ± 0.00 ^d^
c17:1n7	0.03 ± 0.00 ^b^	0.03 ± 0.00 ^b^	0.03 ± 0.00 ^a^	0.02 ± 0.00 ^d^	0.03 ± 0.00 ^c^
c18:1n9t	0.01 ± 0.00 ^a^	0.01 ± 0.00 ^a^	0.01 ± 0.00 ^a^	0.01 ± 0.00 ^c^	0.01 ± 0.00 ^b^
c18:1n9c	1.16 ± 0.00 ^ab^	1.14 ± 0.00 ^a^	1.24 ± 0.01 ^bc^	0.93 ± 0.00 ^c^	1.08 ± 0.00 ^bc^
c20:1n9	0.06 ± 0.00 ^a^	0.06 ± 0.00 ^b^	0.07 ± 0.00 ^a^	0.05 ± 0.00 ^d^	0.06 ± 0.00 ^c^
c22:1n9	0.04 ± 0.00 ^b^	0.03 ± 0.00 ^c^	0.04 ± 0.00 ^a^	0.03 ± 0.00 ^e^	0.04 ± 0.00 ^d^
c18:2n6t	0.02 ± 0.00 ^b^	0.02 ± 0.00 ^c^	0.02 ± 0.00 ^a^	0.01 ± 0.00 ^e^	0.02 ± 0.00 ^d^
c18:2n6c	0.18 ± 0.00 ^b^	0.18 ± 0.00 ^c^	0.19 ± 0.00 ^a^	0.14 ± 0.00 ^e^	0.17 ± 0.00 ^d^
c20:2n6	0.01 ± 0.00 ^b^	0.01 ± 0.00 ^c^	0.01 ± 0.00 ^a^	0.01 ± 0.00 ^c^	0.01 ± 0.00 ^a^
c18:3n6	0.00 ± 0.00 ^b^	0.00 ± 0.00 ^c^	0.00 ± 0.00 ^a^	0.00 ± 0.00 ^e^	0.00 ± 0.00 ^d^
c18:3n3	0.02 ± 0.00 ^b^	0.02 ± 0.00 ^b^	0.02 ± 0.00 ^a^	0.01 ± 0.00 ^d^	0.01 ± 0.00 ^c^
c20:3n6	0.07 ± 0.00 ^b^	0.07 ± 0.00 ^b^	0.07 ± 0.00 ^a^	0.05 ± 0.00 ^c^	0.06 ± 0.00 ^bc^
c20:4n6	0.01 ± 0.00 ^a^	0.00 ± 0.00 ^ab^	0.01 ± 0.00 ^a^	0.00 ± 0.00 ^c^	0.00 ± 0.00 ^b^
c20:3n3	0.03 ± 0.00 ^a^	0.03 ± 0.00 ^a^	0.03 ± 0.00 ^ab^	0.03 ± 0.00 ^c^	0.03 ± 0.00 ^b^
c20:5n3	0.01 ± 0.00 ^b^	0.01 ± 0.00 ^ab^	0.01 ± 0.00 ^ab^	0.01 ± 0.00 ^ab^	0.01 ± 0.00 ^a^
c22:2n6	1.29 ± 0.02 ^a^	1.21 ± 0.02 ^b^	1.31 ± 0.06 ^c^	1.00 ± 0.02 ^d^	1.12 ± 0.02 ^b^
C22:6n3	0.40 ± 0.00 ^a^	0.39 ± 0.00 ^b^	0.40 ± 0.00 ^a^	0.34 ± 0.00 ^d^	0.38 ± 0.00 ^c^

^1^ Group 1: dried at 95 °C and 101 kPa; Group 2: dried at 95 °C and 60 kPa; Group 3: dried at 75 °C and 101 kPa; Group 4: dried at 75 °C and 60 kPa; and Group 5: dried at 75 °C and 20 kPa during the second stage. The different lowercase letter in the same row indicates that the means are significantly different at *p* < 0.05.

## Data Availability

The original contributions presented in the study are included in the article, further inquiries can be directed to the corresponding author.
